# 3-Dimensional printing in rehabilitation: feasibility of printing an upper extremity gross motor function assessment tool

**DOI:** 10.1186/s12938-020-00839-3

**Published:** 2021-01-05

**Authors:** Naaz Kapadia, Mathew Myers, Kristin Musselman, Rosalie H. Wang, Aaron Yurkewich, Milos R. Popovic

**Affiliations:** 1 Rehabilitation Sciences Institute, University of Toronto, Toronto, ON Canada; 2 The KITE Research Institute, Toronto Rehabilitation Institute, University Health Network, 550 University Avenue, Toronto, ON M5G 2A2 Canada; 3 Rocket Family Upper Extremity Clinic, Toronto Rehabilitation Institute, University Health Network, 550 University Avenue, Toronto, ON M5G 2A2 Canada; 4 Institute of Biomedical Engineering, University of Toronto, Toronto, ON Canada; 5 Department of Physical Therapy, Rehabilitation Sciences Institute, University of Toronto, Toronto, ON Canada; 6 SCI Mobility Laboratory, Lyndhurst Centre, The KITE Research Institute, Toronto Rehabilitation Institute, University Health Network, Toronto, ON Canada; 7 Department of Occupational Science and Occupational Therapy, University of Toronto, Toronto, ON Canada; 8 Intelligent Assistive Technology and Systems Laboratory, The KITE Research Institute, Toronto Rehabilitation Institute, University Health Network, Toronto, ON Canada; 9 Imperial College London, London, UK

**Keywords:** 3D printing, Rehabilitation, Upper extremity, Stroke, Outcome measure, Assessment, Psychometric properties

## Abstract

**Background:**

Use of standardized and scientifically sound outcome measures is encouraged in clinical practice and research. With the development of newer rehabilitation therapies, we need technology-supported upper extremity outcome measures that are easily accessible, reliable and valid. 3‐Dimensional printing (3D-printing) has recently seen a meteoric rise in interest within medicine including the field of Physical Medicine and Rehabilitation. The primary objective of this study was to evaluate the feasibility of designing and constructing a 3D printed version of the Toronto Rehabilitation Institute-Hand Function Test (TRI-HFT). The TRI-HFT is an upper extremity gross motor function assessment tool that measures function at the intersection of the International Classification of Function’s body structure and function, and activity domain. The secondary objective was to assess the preliminary psychometrics of this test in individuals with stroke.

**Results:**

3D design files were created using the measurements of the original TRI-HFT objects. The 3D printed objects were then compared to the original test objects to ensure that the original dimensions were preserved. All objects were successfully printed except the sponge and paper which required some modification. The error margin for weight of the objects was within 10% of the original TRI-HFT for the rest of the objects. Nine participants underwent the following assessments: the Chedoke Arm and Hand Activity Inventory (CAHAI), Fugl Meyer Assessment-Hand (FMA-Hand), Chedoke McMaster stages of recovery of the arm (CMSA-Arm) and Chedoke McMaster stages of recovery of the hand (CMSA-Hand) and the 3D TRI-HFT for assessment of psychometric properties of the test. The video recorded assessment of the 3D TRI-HFT was used for reliability testing. Construct validity was assessed by comparing the scores on 3D TRI-HFT with the scores on CAHAI, CMSA-Arm, CMSA-Hand and FMA-Hand. The 3D TRI-HFT had high inter-rater reliability (Intra-Class Correlation Co-efficient (ICC) of 0.99; *P* < 0.000), high intra-rater reliability (ICC of 0.99; *P* < 0.000) and moderate-to-strong correlation with the CMSA-Arm, CMSA-Hand and FMA-Hand scores.

**Conclusions:**

The TRI-HFT could be successfully 3D printed and initial testing indicates that the test is a reliable and valid measure of upper extremity motor function in individuals with stroke.

## Background

Use of standardized and scientifically sound outcome measures is highly encouraged in clinical practice and research. A number of guidelines have been developed around use of upper extremity outcome measures in stroke [1, 2]. However, researchers have identified that with the development of newer rehabilitation therapies we need technology-supported upper extremity outcome measures that are easily accessible and can measure change consistently and reliably [3]. The most commonly used upper extremity measures in clinical and research settings for stroke in the International Classification of Function’s (ICF’s) body structure and function (WHO-Chapter 4) and activity level domains are the Fugl Meyer Assessment Upper Extremity (FMA-UE) [4] and the Action Research Arm Test (ARAT) [5], respectively. The ARAT is a time-based activity test and although widely used has several documented limitations [6]. Furthermore, Demers et al. reviewed 15 upper extremity outcome measures assessing arm/hand function at the ICF’s activity level recommended by neurological clinical practice guidelines [7]. These include the Box and Block test [8], Jebsen Hand function test [9], Nine hole peg test [10], ARAT [5], Chedoke Arm and Hand Activity Inventory (CAHAI) [11], Arm Motor ability test [12], Frenchay Arm Test [13], Motor Evaluation Scale for Upper Extremity in Stroke Patients [14], Reaching Performance Scale for Stroke [15], Test d’Évaluation des Membres supérieurs des Personnes Âgées [16], Wolf Motor Function Test [17], ABILHAND [18], Capabilities of the upper extremity [19] and Motor-Activity Log [7]. The review concluded that current activity measures may not distinguish recovery from compensation and do not adequately track changes in movement quality over time. Moreover, most of the above stated outcome measures either lack information about validity, reliability or responsiveness in the stroke population or require an administration time of > 20 min, may require equipment purchase or construction or copyright payment [2].

Thus, there is a need for a tool that can reliably and effectively measure change following rehabilitation interventions such as robot-assisted therapies [20], functional electrical stimulation (FES) therapy [21], mirror therapy [22], brain computer interface-controlled FES therapy [23] and many others. The tool needs to be (1) universally and easily accessible, (2) reliable, (3) valid, (4) feasible to be administered within both clinical and research settings, and (5) virtually requiring no training to be used by rehabilitation professionals and research personnel.

We developed the Toronto Rehabilitation Institute-Hand Function Test (TRI-HFT) [24] an upper extremity gross motor function assessment tool that measures function at the intersection of the ICF’s body structure and function and activity domain. The TRI-HFT evaluation requires participants to manipulate everyday objects using only the affected upper extremity and measures difficulty with manipulation of these objects using the paretic hand. The scoring differentiates between the use of a physiological grasp versus use of compensatory strategies during task execution and virtually no training is required to administer the test. The scoring system requires the participant to reach for the object, grasp it, lift off the supporting surface and finally manipulate it before replacing it back on the table. The original TRI-HFT test consists of two parts; (1) object manipulation and (2) strength measurement. The object manipulation part of the test consists of day-to-day objects that are manipulated using different types of grip. The test objects include: (1) mug, (2) paper, (3) book, (4) Ziploc bag filled with five golf balls, (5) pop can, (6) dice, (7) isosceles triangular sponge, (8) credit card, (9) wireless home telephone, (10) pencil and (11) nine rectangular blocks in sets of 3 × 100 g, 3 × 200 g and 3 × 300 g. Each of the three blocks in each weight category have surfaces with different levels of friction. This part of the test is graded on a 0–7 scale where higher scores indicate better performance. The strength measurement part of the test measures the strength of lateral grip and palmar grasp using three sub-tests, i.e. instrumented credit card, instrumented cylinder, and the wooden bar. It takes approximately 15 min to administer the original TRI-HFT, on bilateral upper extremities, in individuals with spinal cord injury [26]. Administration and scoring of the test do not require special training and can be administered by reviewing a two-page instruction sheet. Details on the original test object dimensions and administration are described in Kapadia et al. [24]. The original version of the test was validated in sub-acute spinal cord injury population, however, was not validated in stroke.

The test, however, failed to see a significant uptake primarily, because it was a challenge to make this test available to interested clinicians and researchers within and outside of Canada. The original TRI-HFT was fabricated by the researchers and while many of the test objects were “off the shelf objects” some were manufactured in our laboratory. Although the original test dimensions are published, we realized that it was challenging for clinicians to compile the test and besides if the test object dimensions changed than standardization of the test would become questionable. This limitation became the motivation for exploring the ability to 3D print the test to make it universally accessible. 3D-printing has recently seen a meteoric rise in interest within medicine, and the field of Physical Medicine and Rehabilitation is no exception [25]. Researchers are discovering many medical and dental applications including devices and implants, biosynthetic and hybrid human tissues including skin, cartilage, and bone and many more applications [26–28]. For the field of physical medicine and rehabilitation, 3D printing has the potential to provide unique solutions to common obstacles related to fabrication and delivery [25]. 3D printing within the rehabilitation world has mostly been explored for fabricating orthosis, prosthesis and for assistive technologies [29–31].

The primary objective of the current study was to assess the feasibility of designing and constructing a 3D printed version of TRI-HFT objects (3D TRI-HFT). It is important to note that the administration of the 3D TRI-HFT and the scoring system remains the same as the original TRI-HFT [26]. The secondary objective was to do a preliminary testing of the psychometric properties of the 3D printed test, specifically the inter and intra-rater reliability of the first part of the test and construct (convergent) validity of the first and second part of the test in individuals with spinal cord injury (under review in The Journal of Spinal Cord Medicine) and chronic stroke. The motivation for testing the 3D printed version of TRI-HFT in stroke was the lack of a stroke-specific measurement tool that is universally easily accessible, can detect change following newer rehabilitation interventions and is able to measure outcomes that are important to stroke survivors and their caregivers.

## Results

**Fig. 1:**
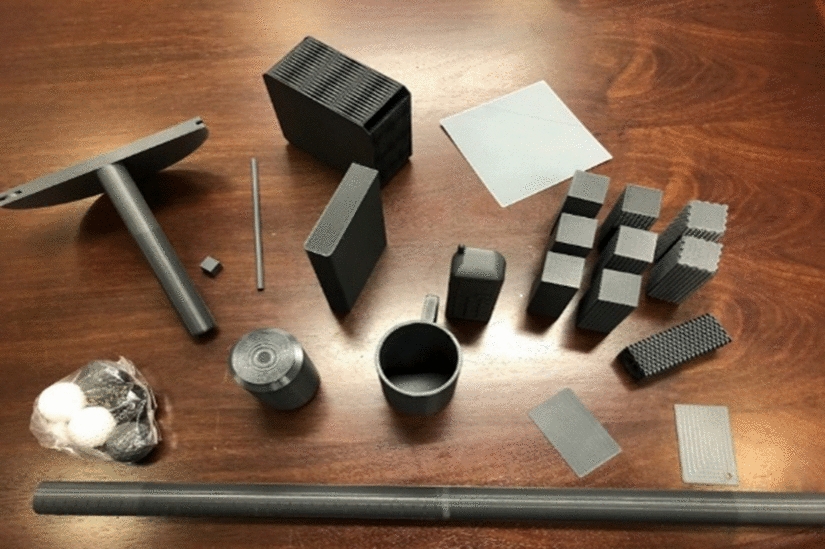
3D printed Toronto Rehabilitation Institute-Hand Function Test (3D TRI-HFT)

The TRI-HFT objects were successfully 3D printed (Fig. 1). Physical dimensions of all objects were within 0.1 mm tolerance except for the sponge and paper, and all weight measurements were within a 10% error margin except for the sponge (Table 1).

**Table 1:** Dimensions of the original TRI-HFT objects and the 3D printed TRI-HFT objects

No.	Object	Measurement of original TRI-HFT objects (size in mm and weight in gm)	Measurement of 3D TRI-HFT objects (size in mm and weight in gm)
1	Mug	Size: 115 × 245	Size: 115 × 250
		Weight: 563.98	Weight: 554
2	Paper	Size: 297 × 210 × 0.1	Size: 150 × 150 × 0.3
		Weight: 0	Weight: 8
3	Book	Size: 173 × 105 × 26	Size: 175 × 105 × 26
		Weight: 315	Weight: 318
4	Zip lock bag with golf balls	Size: 170 × 200	Size: 170 × 200
		Weight: 230 (46 g per ball × 5 golf balls)	Weight: 236 (39 g per ball × 6 golf balls)
5	Pop can	Size:120 × 61	Size:123 × 66
		Weight: 350	Weight: 388
6	Dice	Size: 15 × 15 × 15	Size: 16 × 16 × 16
		Weight: 6	Weight: 4
7	Sponge	Size: isosceles triangle with height 400 and base 200 Weight: 170	Size: Square 142 × 142 Weight: 161
8	Credit card	Size: 85 × 53	Size: 86 × 54 × 08
		Weight: 0	Weight: 4
9	Wireless phone	Size: 144 × 50 × 25	Size: 145 × 50 × 35
		Weight: 223	Weight: 222.3
10	Pencil	Size: 187 × 27	Size: 190 × 27
		Weight: 9	Weight: 6
11	Rectangular blocks	Size: 115 × 35 × 35	Size: 100 × 36 × 36
		Weight: 100/200/300	Weight: 100/200/296
12	Instrumented cylinder	Size: plate radius:110 Handle length:215 Handle circumference: 95 Weight:300	Size: plate radius: 110 Handle length:215 Handle circumference: 95 Weight: 281.49
13	Instrumented credit card	Size: 85 × 53 × 0	Size: 86 × 54 × 08
		Weight: 0	Weight: 4
14	Rod	Size: length:740 Diameter: 33	Size: length:740 Diameter: 33
		Weight: 666	Weight: 642

*mm* millimeters,* gm* grams

The mean time taken to administer the 3D TRI-HFT on the affected upper extremity of the study participants was 10.5 min (Table 2).

**Table 2:** Time taken to complete the 3D TRI-HFT by individual study participant

Participant ID	Time taken to complete the 3D TRI-HFT testing (in minutes)
P31	4.16
P32	12.04
P33	9.54
P34	Not available
P35	11.43
P36	Not available
P39	9.23
P40	13.43
P42	13.41

Nine participants were recruited for psychometric testing of the 3D TRI-HFT. Participants completed the 3D TRI-HFT, CAHAI, Chedoke McMaster Stroke assessment-Impairment Inventory-stage of arm (CMSA-Arm), Chedoke McMaster Stroke assessment-Impairment Inventory-stage of hand (CMSA-Hand) and Fugl Meyer Assessment-Hand (FMA-Hand) assessments. Participant demographics are listed in Table 3.

**Table 3:** Participant demographics

Participant	Duration post-stroke	Paretic/dominant hand	Gender	Age
P31	26 years, 4 months	L/R	F	71
P32	34 years, 11 months	L/R	M	85
P33	10 months	R/R	M	48
P34	8 years, 1 month	L/R	M	58
P35	2 years, 2 months	L/R	M	65
P36	1 year, 9 months	R/R	M	52
P39	3 years, 10 months	L/R	M	59
P40	16 years, 8 months	L/R	M	35
P42	17 years, 3 months	L/L	F	50

*L* left, *R* right, *F* female, *M* male

Validity testing was performed using nine data sets, however, two of the nine participant videos (P34 and P36) were not available and hence reliability testing was performed on seven data sets. Individual participant raw scores as well as descriptive statistics including means, median, range and percentiles for the CMSA-Arm, CMSA-Hand, FMA-Hand and the 3D TRI-HFT components are summarized in Table 4. The inter and intra-rater reliability for the rectangular block’s component of the object manipulation task could not be assessed as the assessors had difficulty identifying the blocks on the videos based on their weight and texture.

**Table 4:** Participants raw scores on upper extremity outcome measures

Participant	CMSA Arm Range 1–7	CMSA Hand Range 1–7	FMA Hand Range 0–14	CAHAI Range 13–91	3D TRI-HFT (no blocks) Range 0–70	3D TRI-HFT (with blocks) Range 0–133	3D TRI-HFT Rod (range: 1–30 on each side)	3D TRI-HFT instrumented cylinder [Nm] Range 0–50	3D TRI-HFT instrumented credit card [N] Range 0–50
P31	2	2	2	18	10	11	0	0	15
P32	3	3	3	24	41	47	15	21	25
P33	2	2	0	22	10	11	0	0	1
P34	3	3	4	38	60	66	30	12.5	42.5
P35	2	2	2	25	10	11	0	10	35
P36	2	2	2	26	13	14	10	15	37.5
P39	2	2	2	30	10	11	20	29	50
P40	4	3	2	38	26	30	0	5	13
P42	7	2	2	54	45	51	15	8	35
Mean	3	2.33	2.11	30.56	25	28	10.7	11.6	28.2
Median	2	2	2	26	–	–	–	–	–
Range	5	1	4	36	–	–	–	–	–
25th percentile	2	2	2	23	–	–	–	–	–
50th percentile	2	2	2	26	–	–	–	–	–
75th percentile	3.5	3	2.5	38	–	–	–	–	–

*CMSA-Arm* Chedoke McMaster Stroke Assessment (Impairment Inventory-stage of arm), *CMSA-Hand* Chedoke McMaster Stroke Assessment (Impairment Inventory-stage of hand), *FMA-Hand* Fugl-Meyer Assessment- hand, *CAHAI* Chedoke Arm and Hand Activity Inventory, *3D TRI-HFT* 3D printed Toronto Rehabilitation institute-Hand Function test

*Scores on all outcome measures are for the affected upper extremity only except for the CAHAI which involves the use of bilateral upper extremity to perform the test

### Intra-rater reliability

**Fig. 2:**
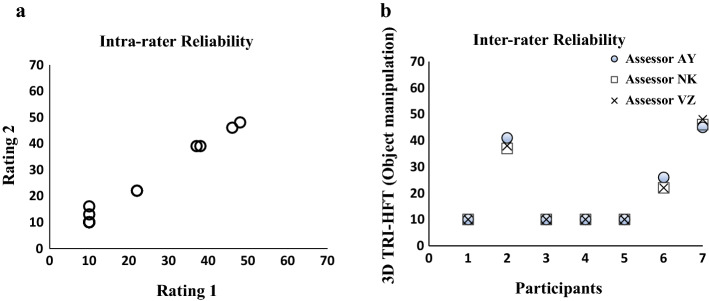
**a** Scoring at two different time points by the same assessor (i.e. rating 1 and rating 2) and **b** scoring by three different assessors at a single time point

There was a high intra-rater reliability for the object manipulation component of the 3D TRI-HFT with an ICC score of 0.99 (95% CI 0.985–0.999; *P* < 0.000) (Fig. 2a).

### Inter-rater reliability

The 3D TRI-HFT was found to have a strong inter-rater reliability for the object manipulation component of the test (Fig. 2b). Intra-class correlation co-efficient (ICC) across three assessors (NK, VZ, AY) was 0.998 (95% CI 0.992–1.0; *P* < 0.000).

### Construct validity

The results showed that there was a statistically significant strong relationship between CMSA-Arm and the object manipulation part of the 3D TRI-HFT (*r* = 0.843, *P* = 0.01). A statistically significant moderate correlation was found between object manipulation of the 3D TRI-HFT and FMA-Hand (*r* = 0.698, *P* = 0.05) and CMSA-Hand (*r* = 0.667, *P* = 0.05). A moderate correlation was found between object manipulation of 3D TRI-HFT and CAHAI (*r* = 0.664, *P* = 0.051), however this correlation was not statistically significant.

For the strength measurement part of the 3D TRI-HFT test, including the rod, the instrumented cylinder and the instrumented credit card, no statistically significant relationship was found with any of the outcome measures, i.e. CAHAI, CMSA-Arm, CMSA-Hand or FMA-Hand. The relationship between the rod and the FMA-Hand approached statistical significance (*r* = 0.665, *P* = 0.051).

## Discussion

3D printing technology has existed for more than 30 years, but has only recently garnered increased attention among scientists, engineers, and the public [32]. The rise of 3D technology is attributed to the availability of lower cost printers and breakthroughs in techniques and processing. In the current study, we established the feasibility of 3D printing the TRI-HFT. We found that all objects of the TRI-HFT could be easily printed except for the triangular sponge and the paper. The discrepancy in dimensions of the paper and sponge are limitations associated with 3D printing process. However, it is important to note that the “paper” and “sponge” created using the 3D technology have the look and design of the original objects and that the modifications are a trade-off that needed to be accepted to make the test universally accessible and reproducible. The sponge required a new design where it will have the properties of the “sponge” but is now shaped as a square instead of a triangle. Today, the TRI-HFT can be 3D printed for ~ CAD $ 500. This cost will decline as 3D printers become cheaper. For reference, the 3D printers costed ~ £175,000 (CAD $300,000)–£250,000 (CAD $ 400,000) in the 1990’s and currently that price has decreased tenfold with high end 3D printers costing ~ £10,000 (CAD $ 20,000)–£35,000 (CAD $ 60,000) [33].

In our convenience sample, of nine individuals with chronic stroke we found that the 3D printed TRI-HFT had high inter- and intra-rater reliability and moderately strong construct validity when compared to the CMSA and FMA. As seen from the results, however, it was not possible to rate the nine rectangular blocks with different weight and textures from the video assessments. These are now modified (numbered) to have a clear indication of which block is being manipulated and this indicator can be seen clearly on video recordings allowing for grading from a video assessment.

Construct validity of the test was assessed as there are no outcome measures identified as a gold standard for measurement of upper extremity function in the activity or body structure and function domain of the ICF [34]. The object manipulation component of the 3D TRI-HFT showed a strong correlation with the CMSA-Arm, whereas there was a moderately strong correlation between 3D TRI-HFT and CMSA-Hand and the 3D TRI-HFT and FMA-Hand. Although the scoring system was initially developed with the spinal cord population in mind, the granular scoring system allows one to capture function equally effectively in stroke population as well. Moreover, the test measures function from proximal to distal and allows the assessor to differentiate between use of physiological versus compensatory grasping patterns. Since most of the study participants had severe upper extremity impairment, distal function was severely compromised and subtle differences went undetected by both the CMSA- hand and the FMA-Hand. The 3D TRI-HFT, however, not only successfully captured arm function as seen by the strong relationship between 3D TRI-HFT and CMSA-Arm but also captured the subtle changes in hand function.

We found no statistically significant relationship between CAHAI and 3D TRI-HFT. This is not surprising given that CAHAI is designed to measure bilateral upper extremity function, whereas 3D TRI-HFT is designed to measure unilateral upper extremity function. In CAHAI, participants can score points by using the non-paretic hand to stabilize the object to assist the paretic hand in grasping an object. Participants can also hold objects with both hands to reduce the gravitational load and use the paretic hand as the supporting hand, while the non-paretic hand performs the accurate arm motions, supinations, and dextrous fine motor skills. Conversely, the 3D TRI-HFT isolates the portions of each task that can be performed unimanually.

We found no statistically significant relationship between the rod, instrumented cylinder and instrumented credit card of the 3D TRI-HFT with any of the other measures. The rod in the TRI-HFT is aimed at measuring the participant’s ability to withstand eccentric forces about the shoulder joint, the instrumented cylinder and the instrumented credit card are both objective measures and they measure torque and grip strength in Nm and N, respectively, using a dynamometer. The scores on these measures are influenced by the strength of the upper extremity muscles and neither the CMSA nor the FMA take into account strength of the muscles but are focused on synergies and movement isolation.

Existing upper extremity measures like the ARAT and the Fugl Meyer Scale commonly used in clinical and research settings fail to meet the demands of these environments, which experience shortages in time and personnel resources. The 3D TRI-HFT takes approximately ~ 11 min to be administered on the affected upper extremity and is cost effective. Another consideration is around importance of the findings of the current assessment tools to patients and their care givers. Stroke survivors identified the outcomes of ‘Independence, freedom and autonomy’, ‘Difficulty (with routine tasks)’ and ‘Everyday tasks’ as their three most important outcomes [35]. The objects used in the 3D TRI-HFT are objects commonly used in activities of daily living. Besides, the scoring system measures the ability to manipulate these objects as they would be manipulated during activities of daily living and hence the score on the TRI-HFT provides an objective measure of patient’s independence with these tasks. A recent review conducted by Miller et al., quoted that though there are a 144 upper extremity outcome measures being used in individuals with stroke [35] none of these assessments measure what is important to stroke survivors, their carers and clinicians [35].There are various other review articles in the literature that have looked at existing measures and most have concluded that there is no consensus amongst clinicians regarding best practices related to use of outcome measures or that existing tools are not sensitive to change in function and do not capture outcomes that are important to stroke survivors, carers and clinicians [3, 7, 35–37].

The 3D printed version of the TRI-HFT was pursued to fill this is gap in literature. 3D printing technology is becoming increasing accessible, affordable, it ensures standardization and reproducibility of the outcome assessment tool. As far as we know the TRI-HFT is the first upper extremity measure that can be 3D printed, and hence can be accessed from anywhere in the world. This is important for clinicians and researchers as it gives them easy access to a reliable and valid tool. Furthermore, the objects used in the test are day to day objects that can also be used as therapy tools by clinicians. From a researcher perspective, having an outcome assessment tool that is easy to manufacture in-house is important to reduce dependencies on high-cost manufacturing and out of country equipment orders. In a review conducted by Galeoto et al., the authors concluded that a universal, validated outcome measure is needed to allow comparisons across practice and recommended that future researchers use a common set of outcome assessments [38].

There are certain limitations to the study, like the small sample size, homogenous study sample with very limited upper extremity function and lack of gold standard outcome measures for this population which prevented a more critical comparison of the 3D TRI-HFT. Nonetheless, this test could be easily 3D printed and was found to be reliable and valid. Future studies will look at expanding the psychometric testing to the acute and sub-acute stroke population as well as those with higher levels of upper extremity function.

## Conclusion

Our findings indicate that TRI-HFT is a 3D printable, simple, reliable, open source, and valid measure that can be accessed from virtually anywhere in the world. The 3D printing of the test guarantees high repeatability in object manufacturing and makes the test available to all users with a 3D printer, which are now becoming ubiquitous. The test can be administered with minimal to no training and produces outcomes that are meaningful not only to the professional community but also to the patients themselves and their care givers.

## Methods

For the purpose of 3D printing the TRI-HFT, the dimensions of the original test objects were measured using calipers (precision level 0.01 mm) and the weight of the objects were measured using a kitchen scale (precision level 0.1 gm). These dimensions were used to create 3D printing design files. Objects were printed on the Stratasys Fortus 450mc in Acrylonitrile Butadiene Styrene (ABS) plastic with a resolution of 10 thou (~ 0.254 mm), with the exception of the golf balls, die, paper, and credit cards, which were printed at a resolution of 5 thou (~ 0.125 mm).The rectangular blocks of 200 g and 300 g were weighted with quarters once printed, in order to reach the mass of the original objects. The 200 g block was weighed with 28 CAD quarters and the 300 g block was weighed with 52 CAD quarters. All printing files for the 3D TRI-HFT will be made available at http://www.kite-uhn.com.

Psychometric testing of the 3D TRI-HFT was conducted in both spinal cord injury (under review in The Journal of Spinal Cord Medicine) and stroke population. A cross-sectional single-site trial was conducted to assess the reliability and validity of the 3D TRI-HFT in stroke. The study was conducted at an outpatient clinic in a rehabilitation hospital. The psychometric properties of the 3D TRI-HFT were tested within a pilot study aimed at creating a novel and usable robotic orthosis to improve performance on functional tasks in individuals with chronic stroke. Research Ethics Board approval for this study was obtained along with the approval for the pilot study. The inclusion criteria were: (a) stroke survivors greater than 7 days post-stroke (b) moderate-to-severe Stage of Hand 1–4 on the Chedoke McMaster Stroke Assessment (c) able to understand and speak English (d) able to give informed consent (e) able to attend at least one session at the TRI site where the study was conducted (f) passive range of motion of index finger proximal interphalangeal joint greater than 45° to allow for fitting the robotic glove. The exclusion criteria were: (a) participants with a severe risk for skin breakdown under applied loads (b) participants who are not able to verbally respond about their level of pain (c) participants with severe pain (above 4 on the Pain Rating Scale) in their more affected fingers during massage or active or passive extension (e.g. arthritis, fracture).

Consenting participants underwent a battery of upper extremity assessments including the CAHAI [11], FMA-Hand [4], CMSA-Arm, and CMSA-Hand [39] and the 3D TRI-HFT. The CAHAI is a validated upper limb measure that uses a seven -point quantitative scale to measure function. The scale assesses functional recovery of the arm and hand after a stroke and has demonstrated a high level of measurement quality and clinical utility [3]. The total score on the CAHAI ranges from 13 to 91. Similarly, the FMA-Hand has also demonstrated a high level of measurement quality and clinical utility and is recommended for assessment of upper extremity function in research and clinical practice [3]. The CMSA-Arm and CMSA-Hand is an assessment tool used to measure physical impairment and it assesses stages of recovery of arm and hand on a seven-point scale.

All participants completed all the above measures in one session with and without the robotic glove and were rated by the assessor (AY). To address, inter and intra-rater reliability, and construct validity, participant performance on the first component of the 3D TRI-HFT, i.e. object manipulation without the robotic glove, was video recorded. Prior to the start of the study the assessor (AY) was introduced to the 3D TRI-HFT and received a one-time 30 min training session on how to administer and score the test. The 3D TRI-HFT video recorded assessments were reviewed by two independent assessors (NK—Physiotherapist and VZ—Physician) for intra-rater reliability testing. Inter-rater reliability was assessed between all three assessors (NK, VZ, AY). Construct validity was assessed by comparing scores on the 3D TRI-HFT with the scores on CAHAI, FMA-Hand and CMSA.

### Statistical analysis

All statistical procedures were conducted using SPSS version 16 (SPSS, Inc., USA). A *P* value of significance was set at < 0.05. The following statistical procedures were used for reliability and validity testing:

#### Inter-rater reliability

For the purpose of inter-rater reliability, scores by all three assessors (AY, NK, VZ) were compared using the ICC (*n* = 7). Two participant videos were not available for testing.

#### Intra-rater reliability

For the purpose of intra-rater reliability, assessors NK and VZ reviewed the participant videos 1 month apart and re-rated the performance on individual tasks (*n* = 14). The scores from the two time points were compared using the ICC. Two participant videos were not available for testing.

#### Construct (convergent) validity

The construct validity of the 3D TRI-HFT was established by computing Spearman’s correlation coefficient with the CAHAI, CMSA-Arm, CMSA-Hand and the FMA-Hand scores (*n* = 9). An *r* value of 0.0–0.4 was considered as weak correlation, 0.4–0.7 was considered as moderate correlation, and above 0.7 was considered as strong correlation [40].

## Data Availability

The datasets used and/or analysed during the current study are available from the corresponding author on reasonable request.

## Abbreviations

ICF: International Classification of Function
FMA-UE: Fugl Meyer Assessment Upper Extremity
ARAT: Action Research Arm Test
CAHAI: Chedoke Arm and Hand Activity Inventory
TRI-HFT: Toronto Rehabilitation Institute-Hand Function Test
3 D printing: 3‐Dimensional printing
3D TRI-HFT: 3D printed version of Toronto Rehabilitation Institute-Hand Function Test
CMSA- Arm: Chedoke McMaster Stroke assessment-Impairment Inventory-stage of arm
CMSA- Hand: Chedoke McMaster Stroke assessment-Impairment Inventory-stage of hand
FMA-Hand: Fugl Meyer Assessment-Hand
ICC: Intraclass correlation coefficient
